# A Closed Loop Audit of DEXA Scan Compliance in a Tier 4 Specialist Eating Disorder Unit

**DOI:** 10.1192/bjo.2025.10628

**Published:** 2025-06-20

**Authors:** Chisom Madu

**Affiliations:** North East London Foundation Trust, London, United Kingdom. Cygnet Hospital Ealing, London, United Kingdom

## Abstract

**Aims:** This closed-loop audit aimed to evaluate adherence to NICE guidelines for DEXA scans in a Tier 4 Specialist Eating Disorder Unit and assess the impact of structured recommendations to address gaps in care, including improved integration of scans into protocols, better documentation, and enhanced patient education.

**Methods:** Clinical notes of 54 patients admitted over two years were reviewed against six key standards derived from NICE guidelines. These guidelines recommend DEXA scans for individuals with anorexia nervosa after 9–12 months of illness, with follow-ups every two years while the eating disorder remains active. Scans should be interpreted by qualified professionals, with patients educated on maintaining a healthy BMI as the primary means of improving bone health. Initial findings informed the development and implementation of recommendations focused on integrating DEXA scans into standard admission protocols, improving documentation, and ensuring multidisciplinary collaboration for interpreting results and educating patients. A re-audit conducted seven months later assessed the impact of these interventions.

**Results:** Baseline compliance was poor: only 30% of eligible patients underwent scans, 6% had results explained by professionals, and patient education was documented in 50% of cases. Following the implementation of recommendations:

62.5% of eligible patients received scans, with gaps due to incomplete documentation or unperformed tests.

80% had results interpreted and explained, with pending results accounting for remaining gaps.

Patient education documentation increased to 80%, and adherence to scanning intervals remained at 100%.

However, no documentation was found on hormonal treatments or discussions on scan utility prior to use.

**Conclusion:** The structured recommendations significantly improved compliance with NICE guidelines, streamlining care processes and enhancing multidisciplinary collaboration. Future efforts should address documentation of scan utility and hormonal treatments while continuing audits to sustain progress and ensure high-quality care for individuals with eating disorders.

